# Discovery of Widespread Migrasome Formation During Amoeboid Migration in *Dictyostelium discoideum*

**DOI:** 10.17912/micropub.biology.001935

**Published:** 2026-01-26

**Authors:** Bridget K Plude, Cynthia K Damer

**Affiliations:** 1 Biology Department, Central Michigan University, Mount Pleasant, MI, US

## Abstract

Migrasomes are organelles that form along retraction fibers of migrating cells and mediate intercellular communication. We performed localization studies on the calcium-dependent phospholipid-binding protein, copine E (CpnE), in *Dictyostelium discoideum* . We observed GFP-CpnE labeled retraction fibers and migrasomes in multiple contexts: chemotaxis toward folate, random migration, and early development. Migrasome membranes were stained with WGA and some migrasomes were stained with SYTO RNASelect, indicating the presence of RNA. Our findings suggest that CpnE may have a role in migrasome formation and indicate that migrasomes may play important roles in *Dictyostelium* development, cell communication, and/or homeostasis in a variety of environments.

**Figure 1. Migrasomes and retraction fibers form under three different migratory conditions in Dictyostelium discoideum:**
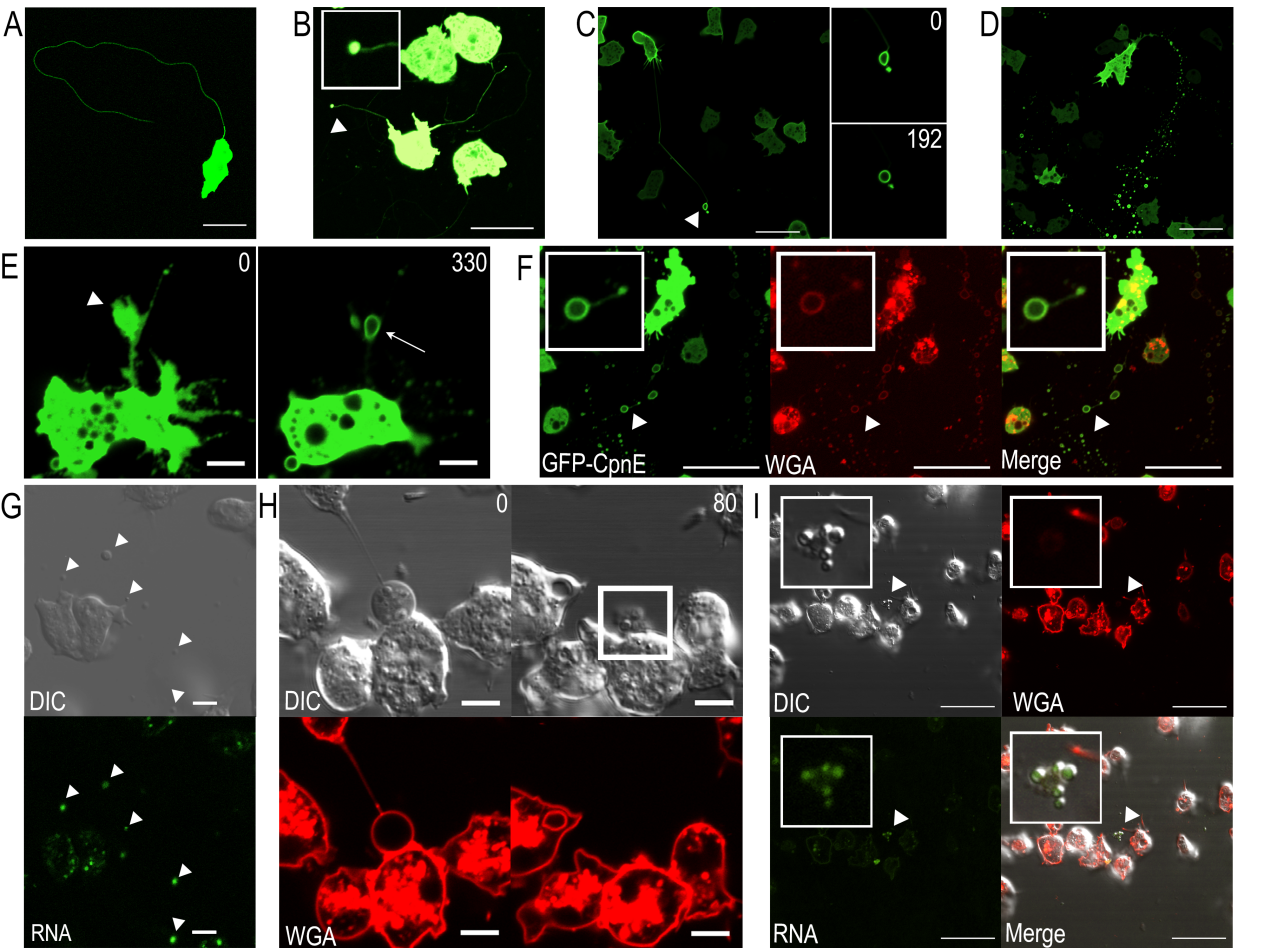
A) NC4A2/GFP-CpnE cells crawling under agar towards folate on BSA coated glass. Scale bar: 20 µm. B) Vesicular structure (arrowhead) at the end of a trail made by NC4A2/GFP-CpnE cells crawling under agar towards folate (inset shows enlarged view of vesicle). Scale bar: 20 µm. C) NC4A2/GFP-CpnE cells migrating randomly on untreated glass. Arrowhead points to GFP-CpnE on the membrane of a migrasome. Scale bar: 10 µm. Time-lapse enlarged images of the migrasome are to the right. Time (s) is indicated. D) NC4A2/GFP-CpnE cell aggregating in development buffer (DB) on untreated glass. Scale bar: 20 µm. E) Time-lapse of NC4A2/GFP-CpnE cells aggregating on untreated glass. Arrowhead points to large structure extending from a retraction fiber (left image). Arrow points to migrasome with GFP-CpnE on the membrane (right image). Time (s) is indicated. Scale bar: 5 µm.&nbsp; F) NC4A2/GFP-CpnE cells aggregating on untreated glass in DB containing WGA at 25 µg/mL. Arrowhead highlights the migrasome seen in upper left insets. Scale bar: 20 µm. G) NC4A2 cells aggregating on untreated glass in DB containing SYTO RNASelect at 1µM. Arrowheads point to migrasome structures with RNA. Scale bar: 5 µm.&nbsp; H) Time-lapse of NC4A2 cells aggregating on untreated glass in DB containing WGA at 25 µg/mL. White box highlights migrasome contents after rupturing out of the plane of view. Time (s) is indicated. Scale bar: 5 µm. I) Inner vesicles inside migrasome released onto untreated glass in DB containing WGA at 25 µg/mL and SYTO RNASelect at 1µM. Scale bar: 2 µm.

## Description

Migrasomes are vesicular organelles that form along retraction fibers of migrating cells (Ma et al. 2015). They contain multiple smaller vesicles carrying diverse cargo such as damaged mitochondria, autophagosomes, signaling molecules, and mRNA (Jiang et al. 2019; Jiao et al. 2021; Zhu et al. 2021; Lee et al. 2024). Migrasomes are released from retraction fibers in a process known as migracytosis and contribute to intercellular communication, morphogen transfer during development, mitochondrial quality control, and immune cell guidance in animals (Lim et al. 2015; Ma et al. 2015; Jiang et al. 2019; Jiao et al. 2021). Migrasomes are also made by cancer cells, where they promote tumor growth and metastatic spread (Zhang et al. 2024; Huang et al. 2025; Wang et al. 2025). A recent study confirmed their presence in the amoeba, *Dictyostelium discoideum* , forming when cells migrate on highly adhesive surfaces (Yu et al. 2025). Here, we expand on migrasome research in&nbsp; *Dictyostelium* &nbsp;by identifying additional migration processes involving these organelles.

*Dictyostelium* lives in the soil as single amoebae feeding on bacteria when food is available but develops into multicellular structures when starved. In both states, the cells use chemotaxis: to follow folate released by bacteria during feeding and to follow cAMP signals released by other cells during development (Kessin 2001). We are using *Dictyostelium* to study a conserved family of calcium-dependent phospholipid-binding proteins called copines. This study focuses on Copine E (CpnE), one of six copine proteins identified in *Dictyostelium* . In a previous study, we showed that GFP-tagged CpnE is a soluble cytosolic protein that binds strongly to phosphatidylserine and phosphatidylinositol-4-phosphate in the presence of calcium. In starved cells, GFP-CpnE exhibited a rapid and robust translocation from the cytosol to the plasma membrane in response to cAMP stimulation. This response was distinct from the other copine family members examined, which exhibited slower kinetics, incomplete translocation, and less sustained membrane association after cAMP stimulation (Ilacqua et al. 2018), suggesting CpnE may have a unique role in cAMP chemotaxis. While studying CpnE localization during chemotaxis, we discovered structures consistent with migrasomes.

Cells expressing GFP-tagged CpnE (GFP-CpnE) were used in an under-agar folate chemotaxis assay and visualized with confocal microscopy. As cells migrated, they produced long, thin GFP-CpnE labeled trails ( Figure 1A ). At the end of trails, there were often small GFP-CpnE labeled vesicular structures that remained stationary while the cell continued migrating ( Figure 1B, arrowhead, inset). At the time of our initial observations, migrasomes had not been reported in&nbsp; *Dictyostelium* . However, because they showed striking similarities to retraction fibers and migrasomes reported in animal cells, we hypothesized that these structures were indeed migrasomes.

Next, we asked whether these structures formed when cells were randomly moving in media on glass, without physical compression. Again, we observed what we believed were retraction fibers and migrasomes; however, the migrasomes appeared larger. Due to the larger size, we were able to visualize that CpnE was associated with the membrane of the migrasomes ( Figure 1C, arrowhead). Using time-lapse imaging, we were able to capture the formation of a migrasome as it became more circular over time ( Figure 1C, right images).

We then asked if cells made migrasomes during aggregation, the first stage of development. Aggregating cells produced migrasomes that were often found in clusters or in a chain marking a migration path ( Figure 1D ). Time-lapse imaging captured a large bulbous structure extending off a retraction fiber ( Figure 1E, arrowhead) that transformed into a ringlike structure ( Figure 1E, white arrow) over time, indicating CpnE recruitment from the cytosol to the membrane. Next, we used wheat germ agglutinin (WGA) to label migrasomes during aggregation. WGA labels glycoproteins and glycolipids found on the extracellular surface of membranes and has been routinely used to identify migrasomes and distinguish them from non-membranous structures and cellular debris (Chen et al. 2019). The GFP-CpnE signal and WGA stain colocalized on migrasome membranes ( Figure 1F, insets). &nbsp;

To determine if *Dictyostelium* migrasomes contain mRNA, we used cells that were not expressing GFP-CpnE and stained them with SYTO RNASelect and imaged cells with differential interference contrast (DIC) and confocal microscopy. We found examples of both large and small migrasomes that were labeled with SYTO RNASelect ( Figure 1G, arrowheads). We also used time-lapse imaging to capture a large migrasome that ruptured ( Figure 1H ). Once ruptured, the migrasome membrane was no longer stained with WGA ( Figure 1H, white box). By refocusing on the expelled contents, we found that the inner vesicles were stained with the SYTO RNASelect ( Figure 1J ), indicating the presence of RNA.

Our novel findings demonstrate that migrasome formation occurs under three different migratory conditions in *Dictyostelium* : chemotaxing under agarose towards folate, migrating randomly in media on glass, and aggregating under starvation conditions on glass. Retraction fibers and migrasomes were observed in all cases. However, retraction fiber length and migrasome number and size varied. The longest retraction fibers and smallest migrasomes were observed in cells migrating under agar. We hypothesize agar compression may disrupt migrasome formation, which is why the migrasomes were smaller in the under-agar experiments. When allowed to freely form in random migration and aggregation, the retraction fibers were more difficult to image because they were able to float away from the glass, while the migrasomes were able to grow and stabilize over time. Notably, cells that were not expressing GFP-CpnE appeared to make the largest migrasomes ( Figure 1H ). We did not observe migrasomes associated with every cell during imaging experiments, and because migrasomes are released from retraction fibers, it was often difficult to link individual migrasomes to their cell of origin. Future studies will examine the factors governing migrasome formation and why only some cells are observed with associated migrasomes within a given field of view.

Our observations indicate that migrasome formation in&nbsp; *Dictyostelium* &nbsp;occurs during early aggregation. In this context, migrasomes may act as concentrated signal packages, carrying mRNA or signaling molecules to enhance intercellular coordination. Alternatively, they may serve a homeostatic role, allowing cells to dispose of unnecessary or damaged components during the developmental transition. The enrichment of CpnE on these structures suggests CpnE could have a role in migrasome formation, perhaps by selecting cargo or enhancing vesicular stabilization. Calcium has been shown to be essential for migrasome formation and Synaptotagmin-1, a protein with two C2 domains like copines, is critical for the formation of migrasomes (Han and Yu 2024). Functional studies using *cpnE* mutant cells will be required to determine whether CpnE plays a critical role in migrasome biogenesis and/or function. Our results point to a previously uncharacterized vesicular pathway in&nbsp; *Dictyostelium* , potentially important for development, communication, and/or homeostasis.

## Methods

*Cell Culture*

The *Dictyostelium discoideum* strain used was NC4A2, an axenic strain made from the NC4 strain (Knecht and Shelden 1995) and was obtained from the Dicty Stock Center (Fey et al. 2013). Cells were grown in VL6 medium (Formedium, VL60102) supplemented with penicillin-streptomycin at 60 U/mL at 18°C on plastic Petri dishes. The cDNA for *cpnE* was subcloned into the *SacI* site of the pTX-GFP plasmid (Levi et al. 2000; Ilacqua et al. 2018). The plasmid was electroporated into NC4A2 cells; cells were pulsed twice with a 5 second interval at 0.85 kV. &nbsp;Cells were placed on ice for 5 minutes and then plated in VL6. Transformants (NC4A2/GFP-CpnE cells) were selected using G418 (geneticin) at 60 µg/mL after 24 hours. Expression of GFP-CpnE was verified with a Western blot using an α-GFP antibody (Ilacqua et al. 2018; Santa Cruz sc-9996).

*Under Agar Chemotaxis Assay*

Glass-bottom plates (35 mm) were incubated with BSA (10% in water) for 8 minutes, rinsed, and allowed to dry. Agarose (1%) was added to SM media (1% glucose, 1% proteose peptone, 0.1% yeast extract, 0.05% MgSO _4_ , 0.19% KH _2_ PO _4_ , 0.06% K _2_ HPO _4_ , pH 6), melted, and then 2 mL was added to each plate. After solidification, a rectangular trough in the center of the plate was cut out of the agarose and two holes were made 5 mm from the trough with a plastic straw. NC4A2/GFP-CpnE cells were harvested from plates and centrifuged at 1500 rpm for 5 minutes. Cells were resuspended in SM media at 5x10 ^6^ cells/mL and 30 µL was added to each hole in the agarose plate. A 100 mM folic acid stock was prepared and 100 µL of the stock was added to 900 µL of warmed SM agarose. The mixture was added to fill the rectangular trough. A coverslip was placed over the center of the plate to cover holes. Migrating cells were imaged 2-8 hours later.

*Random Movement Assay*

NC4A2/GFP-CpnE cells were harvested from plates, counted using a hemocytometer, and centrifuged at 1500 rpm for 5 minutes. The cells were resuspended in VL6 at 2x10 ^6^ cells/mL. In 35 mm glass bottom dishes, 1 mL of cells was mixed with 1 mL of VL6. Cells were incubated at room temperature for 5-6 hours. Immediately before imaging, VL6 was replaced with developmental buffer ((DB) 5 mM Na _2_ HPO _4_ , 5 mM KH _2_ PO _4,_ pH 6.5).&nbsp;

*Streaming Assay*

NC4A2 and NC4A2/GFP-CpnE cells were harvested from plates, counted using a hemocytometer, and centrifuged at 1500 rpm for 5 minutes. Cells were resuspended in DB at 5x10 ^6^ &nbsp;cells/ml. In 35 mm glass bottom dishes, 1 mL of cells was mixed with 1 mL of DB. Cells were incubated at room temperature for 6 hours. Prior to imaging, DB on plates with NC4A2/GFP-CpnE was replaced with DB containing wheat-germ agglutinin (WGA) Alexa Fluor 555 (Invitrogen W32464) at 25 µg/mL. On plates with NC4A2 cells, DB was replaced with DB containing WGA at 25 µg/mL and/or SYTO RNASelect (Invitrogen S32703) at 1µM.

*Confocal Imaging and Analysis*

All images were taken with a Nikon A1R laser scanning confocal microscope. GFP-CpnE, WGA-Alexa Fluor 555, and SYTO RNASelect were detected using 488, 561, and 488 nm lasers, respectively. Cells were imaged using the Plan Apo 60X oil-immersion objective with a 1.2 NA. Laser power was consistent between imaging at 8.00. Images were collected at 1024 x 1024 pixels. Image averaging was set to 4X, 8X, or 16X to reduce background noise. HV and offset values were utilized to optimize individual images.

## Reagents

**Table 1. Cell Strains and Plasmids**

**Table:** 

Cell strain /plasmid name	Description	Origin	Available from
NC4A2	Axenic *Dictyostelium* Strain	Knecht & Sheldon 1995	Dicty Stock Center
pTX-GFP/CpnE	Extrachromosomal vector (pTX-GFP) with *cpnE* cDNA inserted at the *SacI* site	Levi et al. 2000 (pTX-GFP); Ilacqua et al. 2018 (pTX-GFP/CpnE)	This lab

**&nbsp;**
